# Identification of C9-C11 unsaturated aldehydes as prediction markers of growth and feed intake for non-ruminant animals fed oxidized soybean oil

**DOI:** 10.1186/s40104-020-00451-4

**Published:** 2020-05-08

**Authors:** Jieyao Yuan, Brian J. Kerr, Shelby M. Curry, Chi Chen

**Affiliations:** 1 Department of Food Science and Nutrition, University of Minnesota, 1334 Eckles Avenue, St. Paul, MN 55108 USA; 2 USDA-ARS National Laboratory for Agriculture and the Environment, 1015 N. University Boulevard, 2165 NSRIC, Ames, IA 50011 USA; 3 Oak Ridge Institute for Science and Education, Oak Ridge, TN USA

**Keywords:** Aldehydes, Broiler, Growth performance, Pig, Thermally oxidized soybean oil

## Abstract

**Background:**

The benefits of using the oxidized oils from rendering and recycling as an economic source of lipids and energy in animal feed always coexist with the concerns that diverse degradation products in these oxidized oils can negatively affect animal health and performance. Therefore, the quality markers that predict growth performance could be useful when feeding oxidized oils to non-ruminants. However, the correlations between growth performance and chemical profiles of oxidized oils have not been well examined. In this study, six thermally oxidized soybean oils (OSOs) with a wide range of quality measures were prepared under different processing temperatures and processing durations, including 45 °C-336 h; 67.5 °C-168 h; 90 °C-84 h; 135 °C-42 h; 180 °C-21 h; and 225 °C-10.5 h. Broilers and nursery pigs were randomly assigned to diets containing either unheated control soybean oil or one of six OSOs. Animal performance was determined by measuring body weight gain, feed intake, and gain to feed ratio. The chemical profiles of OSOs were first evaluated by common indicative tests, including peroxide value, thiobarbituric acid reactive substances, *p*-anisidine value, free fatty acids, oxidized fatty acids, unsaponifiable matter, insoluble impurities, and moisture, and then analyzed by the liquid chromatography-mass spectrometry-based chemometric analysis.

**Results:**

Among common quality indicators, *p*-anisidine value (AnV), which reflects the level of carbonyl compounds, had the greatest inverse correlation with the growth performance of both broilers and pigs, followed by free fatty acids and oxidized fatty acids. Among the 17 aldehydes identified in OSOs, C9-C11 alkenals, especially 2-decenal and 2-undecenal, had stronger inverse correlations (*r* < − 0.8) with animal performance compared to C5-C8 saturated alkanals, suggesting that chain length and unsaturation level affect the toxicity of aldehydes.

**Conclusions:**

As the major lipid oxidation products contributing to the AnV, individual C9-C11 unsaturated aldehydes in heavily-oxidized oils could function as effective prediction markers of growth and feed intake in feeding non-ruminants.

## Background

Lipids are crucial components in the feeds for non-ruminants production, functioning to increase caloric density, provide essential nutrients, and enhance palatability [1, 2]. Besides the oils obtained through oilseed processing, extracted lipids from fruits or nuts, animal rendering, and restaurant oil recycling are also economic sources of lipids for animal feed formulation. For example, rendering industry in the United States produced around 9,253,000 metric tons of rendered fats and recycled oils in 2010, of which about 7% of these products were used in animal feeds [3]. Despite the availability and economy of these extracted or processed lipids, there remain concerns on their quality and nutritional value due to the thermal processing in preparation and the temperature during storage [4]. The temperature of stored bulk oil can range from 45 to 60 °C depending upon the type of lipids used in feed formulation and the location of the feed production facility [5]. The temperature of steaming and heating used for fat separation and sanitation of animal byproducts in the rendering industry can range from 115 to 145 °C [6]. In deep frying cooking, the temperature adopted by restaurants and food industries generally ranges from 175 to 190 °C, while baking and pan frying can go over 225 °C. Under these thermal treatments, hydrolysis, oxidation, and polymerization of fat or oil can occur, resulting in the degradation of triacylglycerols, fatty acids, and antioxidants, as well as the formation of lipid oxidation products (LOP) [7]. Aldehydes, including *α, β*-unsaturated aldehydes, are a major group of bioactive secondary LOP that have been shown to cause cytotoxicity and genotoxicity at high doses [8]. Long-term exposure of *α, β*-unsaturated aldehydes, especially 4-hydroxynoneal (4-HNE), have also been associated with adverse health effects [9–13]. Interestingly, thermally oxidized oils also contain the ligands of peroxisome proliferator-activated receptor α (PPARα), which can decrease triacylglycerol and cholesterol levels in the liver and plasma for the prevention of dyslipidemia-related morbidities [14–18]. All these bioactivities of oxidized oils have potential to affect the growth and feed intake of exposed animals.

In poultry and swine production, feeding oxidized lipids has been shown to reduce growth rate, feed intake, and feed efficiency [16, 19–21]. The quality of lipid ingredients in animal feeds has been commonly evaluated using indicative tests such as peroxide value (PV), thiobarbituric acid reactive substances (TBARS), *p*-anisidine value (AnV), free fatty acids (FFA), oxidized fatty acids (OFA), total polar compounds (TPC), and polymerized tryacylglycerides (PTAG) [16, 22, 23]. The correlations of these lipid quality indicators with animal performance have been examined in feeding trials with marginal success, mainly due to the fact that these trials only evaluated a limited number of fat or oil samples, of which the range of lipid oxidation was not broad enough for robust correlation analysis [20, 24–26]. To address this deficiency by expanding the pool of observations, a meta-analysis on the correlations of PV and TBARS with growth performance in multiple poultry and swine trials has been conducted, but the effort was still confounded by different oil sources and inconsistent oxidation methods examined across the feeding trials [27]. Hence, identification and characterization of robust quality markers that can accurately predict animal performance in feeding oxidized oils is still needed.

In the study reported herein, broilers and nursery pigs were fed with an unheated control soybean oil (CSO) or six different thermally oxidized soybean oils (OSOs). Animal performance was first correlated to the common quality indicators, and then a liquid chromatography-mass spectrometry (LC-MS)-based chemometric analysis was conducted to identify and characterize specific chemical species contributing to the observed correlations.

## Materials and methods

All animal care and use procedures in animal experiments were approved by the Institutional Animal Care and Use Committee at Iowa State University (IACUC # 4-18-8742-GS).

### Oxidized soybean oil generation

Soybean oil with no added antioxidant was purchased in bulk from Stratas Foods (Memphis, TN, USA). OSOs with a wide range in LOP were prepared at 6 different combinations of temperatures and heating durations based on past research with minor modifications [5, 23], including 45 °C-336 h, 67.5 °C-168 h, 90 °C-84 h, 135 °C-42 h, 180 °C-21 h, and 225 °C-10.5 h. Heating was conducted by filling a 140-L aluminum stock pots with 75 L of soybean oil, continuously bubbling the air (30 L/min) into the oil through four 3.2 mm holes. Immersion heaters were used to prepare 45 °C-336 h, 67.5 °C-168 h, 90 °C-84 h, 135 °C-42 h, and 180 °C-21 h oils, while a propane heater for the 225 °C-10.5 h oil. Temperatures in these heating processes were recorded at regular intervals, and were kept close to the desired temperatures (Additional file 1: Table S1). After the preparations, all OSO samples were stored at − 20 °C without adding antioxidant prior to diet mixing.

### Broiler management and diet formulation

Day-old, Ross 708 chicks were obtained from Welp Hatchery (Bancroft, IA), placed into 77 pens, 1.2 m × 1.2 m covered with wood-shavings, and fed a common diet for 7 d. At 7 d of age (day 0 of the experiment), chicks were weighed (initial body weight (BW) 112 ± 5 g) and randomly assigned to one of the 7 dietary treatment groups. Each dietary treatment group consisted of 11 pens with 10 birds per pen. Upon placement, the room temperature was kept at a maximum of 32 °C for the first 7 d and then decreased by about 1 °C per day to a final temperature of 21 °C for the rest of the trial. The lighting schedule was 23 h of light and 1 h of dark for the first 7 d, and then 20 h of light and 4 h of dark afterwards. Diets in the two phases of feeding (phase 1 from d 0 to 14 and phase 2 from d 15 to 28) contain the same levels of lipids (7.5% CSO or OCO), but differ in the concentration of amino acids and macro minerals (Additional file 1: Table S2). All diets met or exceeded industry recommended specifications [28]. Diets were fed in mash form and birds were allowed *ad libitum* access to feed and water during the experiment. Birds and feeders were weighed at the beginning and end of each phase to calculate overall average daily gain (ADG), average daily feed intake (ADFI) and gain to feed ratio (G:F).

### Pig management and diet formulation

Two hundred and thirty one mixed gender Genetiporc 6.0 × Genetiporc F25 pigs (PIC, Inc., Hendersonville, TN, USA) were obtained from a commercial farm at weaning (28 d of age), and transported and housed at the Iowa State University Swine Nutrition Farm. Upon arrival, pigs were group-fed a common starter diet for 7 d to optimize feed intake during the weaning transition period. Afterwards, pigs were moved into an environmentally controlled room and randomly placed into 77 pens (0.9 m × 2.1 m) with 3 pigs per pen, resulting in 11 replications per dietary treatment. Test diets (Additional file 1: Table S2) were formulated to be adequate in energy and nutrients relative to the National Research Council (2012) recommendations [29] and were randomly allotted to the pen. Pigs were allowed *ad libitum* access to feed and water during the experiment. Feeders and pigs were weighed at the beginning (initial BW = 5.9 ± 0.7 kg) and the end (average BW = 12.1 ± 1.7 kg) of the 25 d experimental period to calculate ADG, ADFI, and G:F.

### Chemicals

Triphenylphosphine (TPP), and 2-hydrazinoquinoline (HQ) were purchased from Alfa Aesar (Ward Hill, MA); 2,2′-dipyridyl disulfide (DPDS) from MP Biomedicals (Santa Ana, CA), USA; LC-MS-grade water and acetonitrile from Fisher Scientific (Houston, TX, USA); Hydrochloric acid from RICCA Chemical Company (Arlington, Texas, USA); trichloroacetic acid and 2-thiobarbituric acid from Sigma-Aldrich (St. Louis, MO, USA); and malonaldehyde bis (dimethyl acetal) from Acros Organics (Morris Plains, NJ, USA). Aldehyde standards, including 2-undecenal, 2,4-undecadienal, 2-decenal, 2,4-decadienal, 2-nonenal, 2,4-nonadienal, 2-octenal, 2-heptenal, 2,4-heptadienal, 2-hexenal, 2-pentenal were provided by Bedoukian Research (Danbury, CT, USA). Octanal and hexanal were purchased from Alfa Aesar (Ward Hill, MA, USA); pentanal and nonanal from TCI America (Portland, OR, USA); acrolein from Sigma-Aldrich (St. Louis, MO, USA); 4-hydroxynonenal (4-HNE) from Cayman Chemical (Ann Arbor, MI, USA); and acetone-*d*_*6*_ from Acros Organics (Morris Plains, NJ, USA). Fatty acids standards (C4-C22) were purchased from Nu-Chek Prep, Inc. (Elysian, MN, USA).

### Common oil quality indicators

Oil samples (COS and the 6 OSOs) were analyzed for moisture, insoluble impurities, unsaponifiable matter, FFA, OFA, PV, and AnV by a commercial laboratory (Barrow-Agee Laboritory, Memphis, TN, USA) using the American Oil Chemists’ Society (AOCS) Official Methods [30] Ca 2c-25, Ca 3a-46, Ca 6a-40, Ca 5a-40, G 3-53, Cd 8b-90, and Cd 18-90, respectively. Measurement for TBARS was conducted using a method previously reported in this laboratory [31].

### Aldehyde determination, chemometric analysis, and data visualization

#### Derivatization of aldehydes and free fatty acids in oil samples

Aldehydes and free fatty acids in oil samples were derivatized by HQ prior to the LC-MS analysis [31]. Briefly, 2 μL of oil sample (in triplicate) was added into 70 μL of freshly prepared acetonitrile solution containing 1 mmol/L DPDS, 1 mmol/L TPP, 1 mmol/L HQ, and 50 μmol/L acetone-*d*_*6*_ (internal standard). After the incubation at 60 °C for 30 min, the reaction was terminated by chilling sample on ice and adding 100 μL of H_2_O. After vortexing, the mixture was centrifuged at 12,000×*g* for 10 min, and the supernatant was transferred into a HPLC vial for LC-MS analysis.

#### LC-MS analysis

An ultra-performance liquid chromatography (UPLC) system (Acquity, Waters, Milford, MA, USA) equipped with a BEH C18 column (Waters) and a Xevo-G2-S quadrupole time-of-flight mass spectrometer (QTOFMS, Waters) were used for LC-MS analysis. A 5 μL sample aliquot from HQ derivatization reaction was injected into the UPLC system using a mobile phase gradient (A: H_2_O containing 0.05% acetic acid, v/v, and 2 mmol/L ammonium acetate; B: H_2_O/ACN = 5:95, v/v, containing 0.05% acetic acid, v/v, and 2 mmol/L ammonium acetate) for separation. The LC eluant was introduced into the QTOFMS detector for accurate mass measurement and ion counting. For positive-mode detection, capillary voltage and cone voltage for electrospray ionization were set at 3 kV and 30 V, respectively. Source temperature and desolvation temperature were maintained at 120 °C and 350 °C, respectively. Nitrogen was used as cone gas (50 L/h) and desolvation gas (600 L/h) while argon as collision gas. The collision energy used for tandem MS (MS/MS) fragmentation ranged from 15 to 45 eV. To achieve accurate mass measurement, sodium formate solution with a mass-to-charge ratio (*m/z*) ranging 50–1,200 was used to calibrate the mass spectrometer. In addition, leucine enkephalin ([M + H]^+^ = 556.2771 *m/z*) was injected intermittently in real time to serve as the lock mass. Mass chromatograms and mass spectral data were acquired and processed by MassLynx software (Waters) in centroided mode. The interested compounds were identified by accurate mass measurement, elemental composition analysis, MS/MS fragmentation, and comparisons with authentic standards. The concentration of individual compound was determined by calculating the ratio between the peak area of interested compound and the peak area of internal standard as well as the fitting of the standard curve using QuanLynx software (Waters).

#### Chemometric analysis and data visualization

The procedure of chemometric analysis has been describe previously [31]. The chromatographic and spectral data of oil samples were processed by MarkerLynx software (Waters). The data matrix was exported and visualized by SIMCA-P+ software (Umetrics, Kinnelon, NJ, USA) after converting by *Pareto* scaling. Principal component analysis (PCA) was then conducted to build a model for the data matrix and to separate different samples. The chemical markers contributed to the sample separation were represented in the loadings plot. To define the correlations among chemical markers, hierarchical cluster analysis (HCA) and heat maps were performed according to the relative abundance of identified compounds in samples after Z-score transformation using R program [32].

### Statistical analysis

Growth performance data were analyzed as a completely randomized design with the pen of broilers or nursey pigs as the experimental unit with initial BW serving as a covariate in the pig study. Means were reported and separated using LSMEANS and data was analyzed using Proc MIXED procedure of SAS version 9.4 (SAS Institute, Cary, NC, USA). Correlations between animal growth performance and oil quality markers were evaluated by Pearson correlation using the R program after pretested the correlation pattern. *P* < 0.05 was considered as statistically significant while 0.05 ≤ *P* ≤ 0.10 was considered as a statistical trend.

## Results

### Animal performance

Compared to feeding CSO, feeding the 45 °C-336 h and 67.5 °C-168 h OSOs did not affect the ADG and ADFI of broilers and nursery pigs except a minor effect of the 67.5 °C-168 h OSO on G:F (Table 1). In contrast, feeding the 135 °C-42 h OSO resulted in the lowest ADG, ADFI, and G:F in broilers, and the lowest ADG and G:F in nursery pigs. In broilers, feeding the 180 °C-21 h and 225 °C-10.5 h OSOs decreased ADG, ADFI, and G:F. In nursey pigs, feeding the 90 °C-84 h and 180 °C-21 h OSOs decreased ADG and G:F, while feeding the 225 °C-10.5 h OSO decreased ADG, ADFI and G:F.

**Table 1:** Growth performance of broilers and pigs fed unheated and thermally processed soybean oil

Soybean oil	Broilers^1^			Pigs^2^		
	ADG, g	ADFI, g	G:F	ADG, g	ADFI, g	G:F
22.5 °C-0 h	60.3^a^	83.4^ab^	0.723^a^	282^a^	461^a^	0.613^ab^
45 °C-336 h	60.6^a^	84.3^a^	0.719^a^	285^a^	437^ab^	0.658^a^
67.5 °C-168 h	59.8^a^	84.7^a^	0.712^b^	290^a^	462^a^	0.634^a^
90 °C-84 h	59.4^ab^	84.8^a^	0.700^c^	230^b^	424^ab^	0.544^c^
135 °C-42 h	54.1^d^	78.3^c^	0.691^d^	182^c^	401^b^	0.467^d^
180 °C-21 h	56.2^cd^	80.6^bc^	0.697^cd^	224^b^	414^ab^	0.547^c^
225 °C-10.5 h	57.5^bc^	81.6^b^	0.704^c^	217^b^	390^b^	0.567^bc^
SEM	0.8	1.0	0.003	9.2	19.0	0.022
*P* value	0.001	0.001	0.001	0.01	0.07	0.01

*ADG* average daily gain, *ADFI* average daily feed intake, *G:F* gain to feed ratio

^1^Data represents 11 pens of broilers with 10 birds per pen for each dietary treatment, fed from 7 to 34 days of age, with an average initial and final BW of 112 and 1686 g, respectively

^2^Data represents 11 pens of 3 pigs per pen for each dietary treatment, fed for 25 d, with an average initial and final BW of 5.9 and 12.1 kg, respectively

^a.b.c.d^Different superscripts in a column indicates a difference between treatments (*P* < 0.05 or 0.05 ≤ *P* ≤ 0.10)

### Common quality indicators of OSOs and their correlations with animal performance

General chemical composition and oxidative status of the CSO and the six OSOs were first evaluated by analyzing common quality indicators of edible oils, including moisture, insoluble impurities, unsaponifiable matter, FFA, OFA, PV, TBARS, and AnV. As expected, temperature and duration of thermal treatments had different impacts on these quality indicators (Table 2). Among 6 OSOs, the 67.5 °C-168 h OSO had the highest level of TBARS; the 90 °C-84 h OSO had the highest levels of PV; the 135 °C-42 h OSO had the highest levels of insoluble impurities, FFA, OFA, and AnV; and the 180 °C-21 h OSO had the highest level of unsaponifiable matter (Table 2).

**Table 2:** Common indicators of oil quality for thermally processed soybean oils fed to broilers and pigs

Soybean oil	M, %	I, %	U, %	FFA, %	OFA, %	PV, mEq/kg	TBARS, μmol/L	AnV
22.5 °C-0 h	0.02	0.02	0.42	0.1	0.96	0.4	79.83	1.2
45 °C-336 h	0.02	0.02	0.39	0.1	0.76	17.4	285.48	6.1
67.5 °C-168 h	0.06	0.04	0.63	0.2	4.2	192.8	1014.32	33.1
90 °C-84 h	0.10	0.04	0.49	0.4	1.5	245.0	665.00	172
135 °C-42 h	0.10	0.06	0.32	0.5	8.6	19.3	493.84	441
180 °C-21 h	0.02	0.04	0.69	0.3	7.2	9.6	419.24	416
225 °C-10.5 h	0.02	0.04	0.57	0.3	3.8	3.0	311.47	233

*M* moisture, *I* insoluble impurities, *U* unsaponifiable matter, *FFA* free fatty acids, *OFA* oxidized fatty acids, *PV* peroxide value, *TBARS* thiobarbituric acid reactive substances (malondialdehyde equivalance), *AnV p*-anisidine value (no unit)

**Fig. 1:**
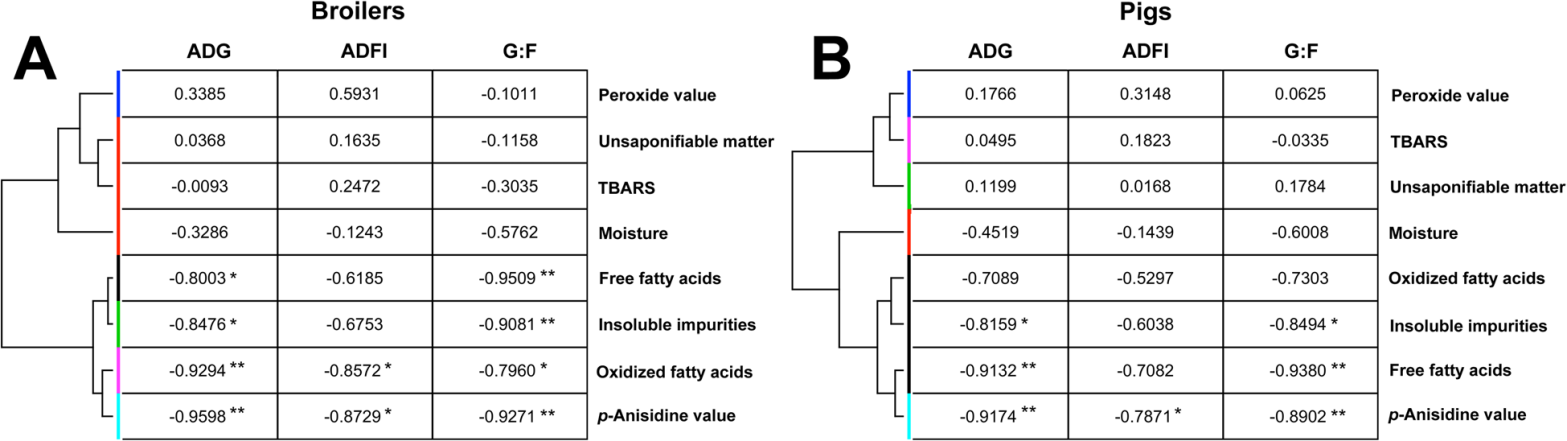
Clustering of common oil indicators based on their Pearson correlation coefficients with animal performance. **a** Correlations in broilers and **b** Correlations in pigs. The values are the Pearson correlation coefficients between individual indicator and individual performance index. *: *P* < 0.05; **: *P* < 0.01

The correlations of these quality indicators with ADG, ADFI, and G:F of broilers and nursey pigs were first evaluated by Pearson correlation analysis, and then characterized by the clustering analysis using the values of their correlation coefficients (Fig. 1a and b). Among these indicators, AnV had the greatest inverse correlations with ADG (*r* = − 0.96, *P* < 0.01), ADFI (*r* = − 0.87, *P* < 0.05), and G:F (*r* = − 0.93, *P* < 0.01) in broilers (Fig. 1a), as well as with ADG (*r* = − 0.92, *P* < 0.01), ADFI (*r* = − 0.79, *P* < 0.05), and G:F (*r* = − 0.89, *P* < 0.01) in nursery pigs (Fig. 1b). FFA, OFA, and insoluble impurities had inverse correlations with specific performance parameters, mainly with ADG and G:F. In contrast, moisture, unsaponifiable matter, PV, and TBARS had poor correlations with the performance of broilers and nursery pigs (0.5931 ≥ *r* ≥ − 0.6008, Fig. 1a and b).

### LC-MS-based chemometric analysis of aldehydes and free fatty acids in OSOs

**Fig. 2:**
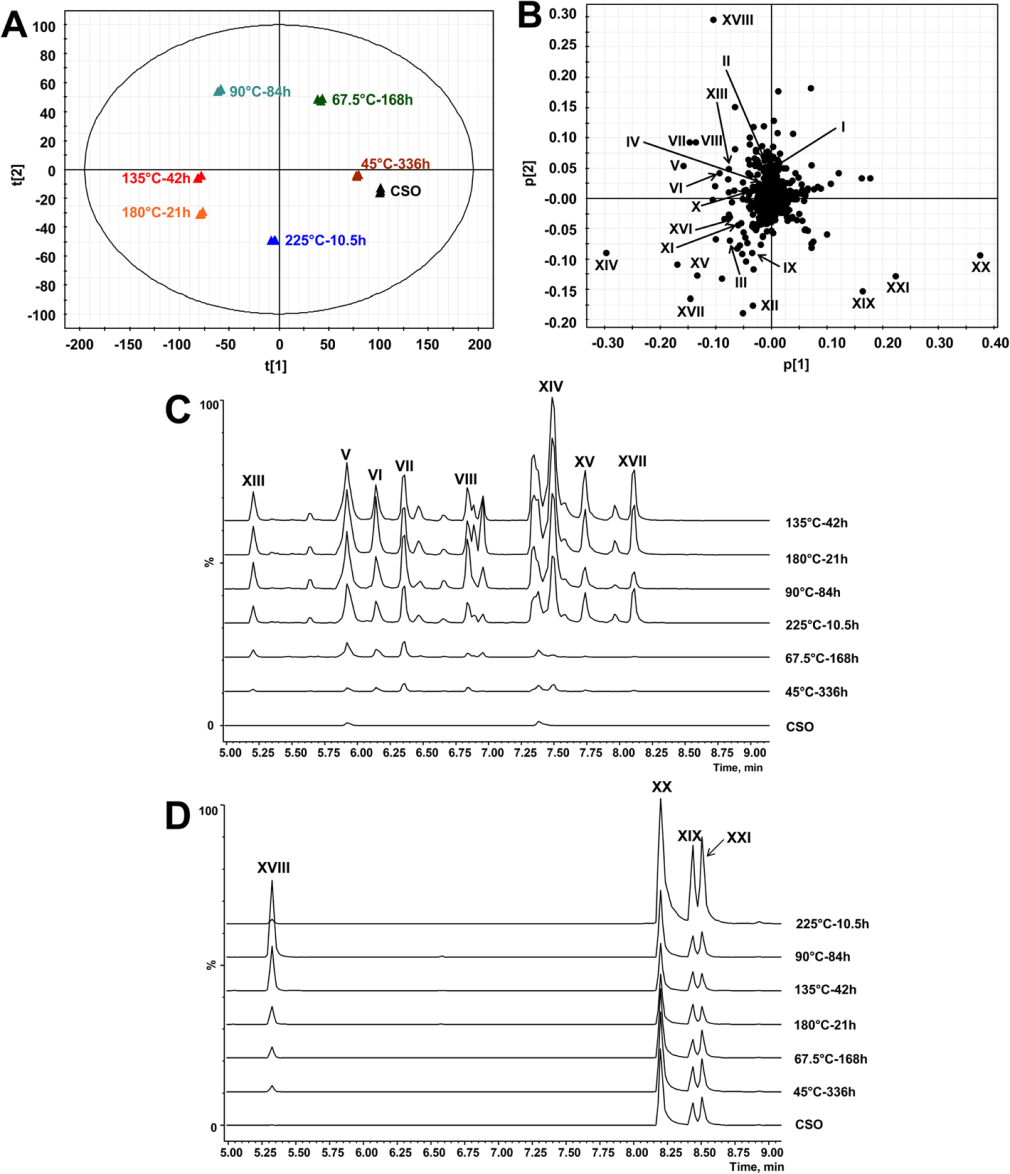
Chemometric model in thermal stress-induced oils (**a**) Scores plot of the PCA model on CSO and OSO samples. The t [1] and t [2] values represent the scores of each sample in the principal components 1 and 2, respectively. **b** Loading plot of the PCA model. Major markers (I-XXI) contributing to sample separation are labeled. The p [1] and p [2] values represent the contributing weights of each ion to the principal components 1 and 2 of the PCA model, respectively. **c** Extracted chromatograms of major aldehyde-HQ derivatives (XIII, V, VI, VII, VIII, XIV, XV, and XVII) in CSO and the 6 OSOs. **d** Extracted chromatograms of major fatty acid-HQ derivatives (XXI, XIX, and XX) in CSO and the 6 OSOs.

**Fig. 3:**
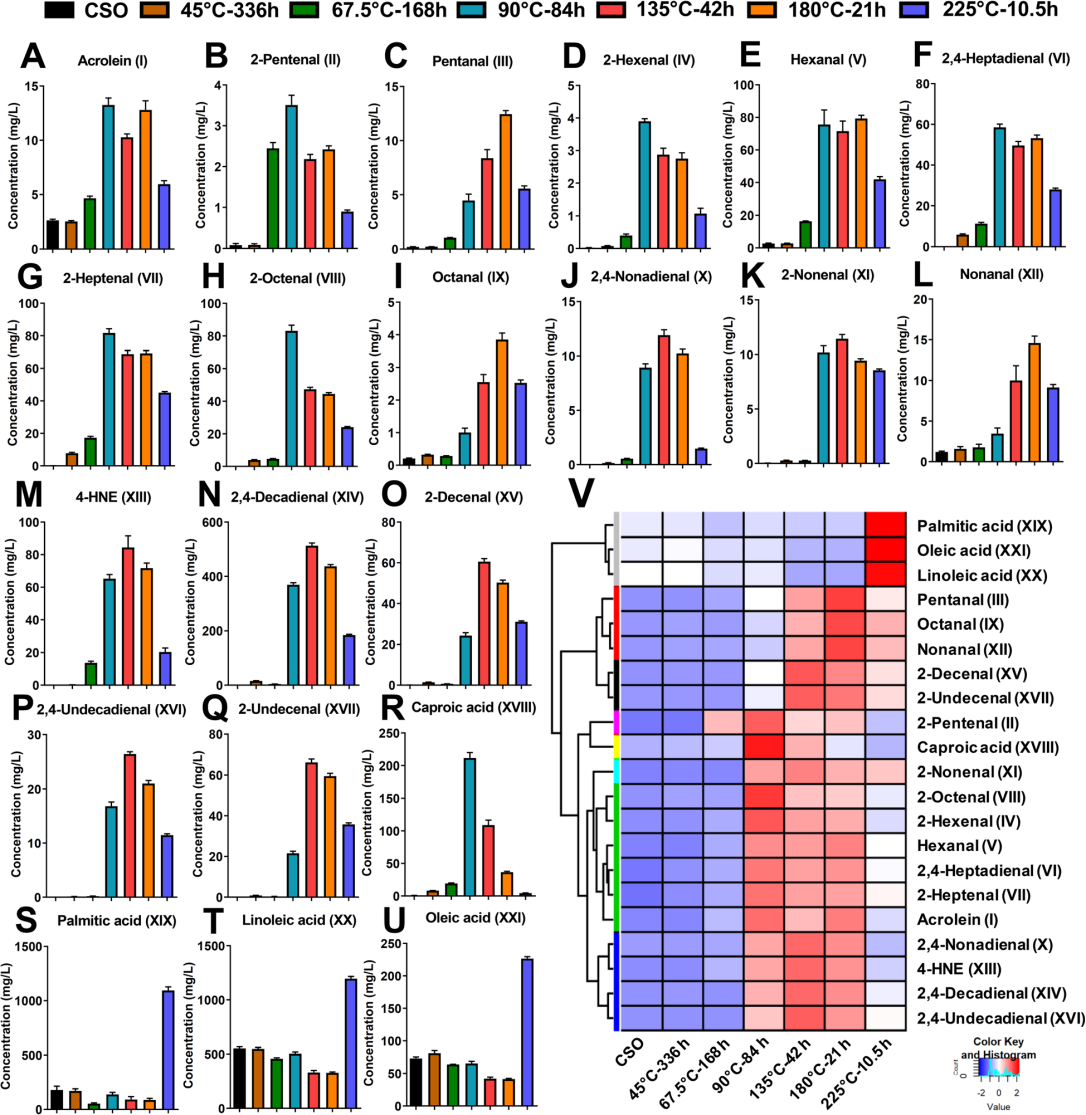
Distribution and concentrations of individual aldehyde and fatty acids markers in CSO and the 6 OSO samples (**a-v**). **a** Acrolein, **b** 2-Pentenal, **c** Pentanal, **d** 2-Hexenal, **e** Hexanal, **f** 2,4-Heptadienal, **g** 2-Heptenal, **h** 2-Octenal, **i** Octanal, **j** 2,4-Nonadienal, **k** 2-Nonenal, **l** Nonanal, **m** 4-HNE, **n** 2,4-Decadienal, **o** 2-Decenal, **p** 2,4-Undecadienal, **q** 2-Undecenal, **r** Caproic acid, **s** Palmitic acid, **t** Linoleic acid, **u** Oleic acid. **v** Heat map and dendrogram of aldehydes and fatty acids from clustering analysis in CSO and OSO samples

In general, AnV, FFA, and OFA are intended to reflect the total concentrations of aldehydes, free fatty acids, and oxidized fatty acids, respectively, but not individual chemical species in oxidized oils. The robust inverse correlations of these three quality markers with animal performance suggest potential contribution of aldehydes as well as free or oxidized fatty acids to the adverse effects of consuming oxidized oils in non-ruminants. However, the roles of individual aldehydes or fatty acid species in the observed correlations cannot be revealed by the values of AnV, FFA, or OFA. Hence, the LC-MS-based chemometric analysis was conducted to define the composition of aldehydes and fatty acids in CSO and the OSOs, as well as their correlations with animal performance. Chromatographic separation and simultaneous detection of diverse aldehydes and fatty acids in the LC-MS analysis were achieved by HQ derivatization, which forms the Schiff bases with the carbonyl group in aldehydes and the hydrazide bond with the carboxyl group in fatty acids, respectively [31, 33]. The changes in the chemical composition of OSOs were characterized by the PCA model of LC-MS data. In the scores plot of a PCA model, the 135 °C-42 h and 180 °C-21 h OSOs were mainly separated from the CSO and the 45 °C-336 h OSO in the 1st principal component (PC) of the model, while 67.5 °C-168 h, 90 °C-84 h, and 225 °C-10.5 h OSOs were mainly separated from others in the 2^nd^ PC of the model (Fig. 2a). This distribution pattern in the PCA model suggests that each heating condition led to distinctive chemical changes in soybean oil. Among the chemical species heavily contributing to the 1^st^ and 2^nd^ PC of the model (Fig. 2b), aldehydes and fatty acids (I-XXI) were confirmed as the main contributors to the separation of oil samples in the PCA model (Table 3). Examining the chromatographs of identified aldehydes in all 7 oil samples showed that the hexanal (V), 2,4-heptadienal (VI), 2-heptenal (VII), 2-octenal (VIII), 4-HNE (XIII), 2,4-decadienal (XIV), 2-decenal (XV), and 2-undecenal (XVII) comprised of the major peaks that were increased by heating, especially high temperature treatments (Fig. 2c). Major free fatty acids identified in the 6 OSOs were caproic acid (XVIII), palmitic acid (XIX), linoleic acid (XX), and oleic acid (XXI) (Fig. 2d). In addition, palmitic acid (XIX), linoleic acid (XX), and oleic acid (XXI) were present in CSO, while caproic acid (XVIII) and the most of identified aldehydes were largely absent in CSO (Fig. 2c and d). Subsequent quantitative analysis of aldehydes and free fatty acids confirmed these observations (Fig. 3a through u), and the distribution pattern of aldehydes and fatty acids in the CSO and the 6 OSOs was further defined by the HCA and the dendrogram branching in a heatmap (Fig. 3v). Three long-chain fatty acids, palmitic acid, oleic acid, and linoleic acid were clustered because of their high abundances in the 225 °C-10.5 h OSO. In contrast, all 17 aldehydes and caproic acid were more abundant in 90 °C-84 h, 135 °C-42 h, or 180 °C-21 h OSOs compared to that in the 225 °C-10.5 h OSO. More specifically, the 180 °C-21 h OSO had the highest concentrations of multiple saturated aldehydes, including pentanal (III), octanal (IX), and nonanal (XII); the 135 °C-42 h OSO had the highest concentrations of multiple dienals, including 2,4-nonadienal (X), 2,4-decadienal (XIV), and 2,4-undecadienal (XVI), as well as 4-HNE (XIII), 2-nonenal (XI), 2-decenal (XV) and 2-undecenal (XVII); the 90 °C-84 h OSO had the highest concentrations of almost all C3-C9 monounsaturated aldehydes as well as caproic acid (XVIII) and 2,4-heptadienal (VI). Overall, the formations of specific aldehydes and fatty acids in OSOs were closely associated with the conditions of thermal treatments.

**Table 3:** Aldehydes and fatty acids in CSO and OSOs^1^

ID	Compounds	Formula	Derivative formula	Calculated exact mass of [M + H]^**+**^	Mass deviation, ppm	Boiling point, °C (predicted/reported)^2^	Log *P*-value^3^
I	Acrolein	C_3_H_4_O	C_12_H_12_N_3_^+^	198.1031	−3.03	52.8 / 52.5	−0.01
II	2-Pentenal	C_5_H_8_O	C_14_H_16_N_3_^+^	226.1344	−1.77	126.8 / 123	1.04
III	Pentanal	C_5_H_10_O	C_14_H_18_N_3_^+^	228.1501	0.44	103.7 / 103	1.44
IV	2-Hexenal	C_6_H_10_O	C_15_H_18_N_3_^+^	240.1501	−0.42	146.5 / 147	1.58
V	Hexanal	C_6_H_12_O	C_15_H_20_N_3_^+^	242.1657	0.41	127.9 / 131	1.97
VI	2,4-Heptadienal	C_7_H_10_O	C_16_H_18_N_3_^+^	252.1501	−0.40	177.4 / 177	1.59
VII	2-Heptenal	C_7_H_12_O	C_16_H_20_N_3_^+^	254.1657	1.18	166.0 / 166	2.11
VIII	2-Octenal	C_8_H_14_O	C_17_H_22_N_3_^+^	268.1814	0.00	190.1 / 171	2.64
IX	Octanal	C_8_H_16_O	C_17_H_24_N_3_^+^	270.1970	−1.11	163.4 / 173	3.03
X	2,4-Nonadienal	C_9_H_14_O	C_18_H_22_N_3_^+^	280.1814	−0.71	222.4 / 222	2.65
XI	2-Nonenal	C_9_H_16_O	C_18_H_24_N_3_^+^	282.1970	0.35	205.0 / 189	3.17
XII	Nonanal	C_9_H_18_O	C_18_H_26_N_3_^+^	284.2127	−0.35	190.8 / 194	3.56
XIII	4-HNE	C_9_H_16_O_2_	C_18_H_24_N_3_O^+^	298.1919	0.34	275.6 / NR^4^	1.85
XIV	2,4-Decadienal	C_10_H_16_O	C_19_H_24_N_3_^+^	294.1970	1.36	244.6 / 244	3.18
XV	2-Decenal	C_10_H_18_O	C_19_H_26_N_3_^+^	296.2127	0.68	230.0 / 230	3.70
XVI	2,4-Undecadienal	C_11_H_18_O	C_20_H_26_N_3_^+^	308.2127	0.00	256.4 / 256	3.71
XVII	2-Undecenal	C_11_H_20_O	C_20_H_28_N_3_^+^	310.2283	1.29	244.8 / 239	4.23
XVIII	Caproic acid	C_6_H_12_O_2_	C_15_H_20_N_3_O^+^	258.1606	0.00	204.6 / NR	1.84
XIX	Palmitic acid	C_16_H_32_O_2_	C_25_H_40_N_3_O^+^	398.3171	0.25	340.6 / NR	7.15
XX	Linoleic acid	C_18_H_32_O_2_	C_27_H_40_N_3_O^+^	422.3171	0.71	360.6 / NR	7.18
XXI	Oleic acid	C_18_H_34_O_2_	C_27_H_42_N_3_O^+^	424.3328	0.00	360.0 / NR	7.70

^1^Aldehydes and fatty acids were detected by HQ derivatization and LC-MS analysis. Structural confirmation was based on accurate mass measurement (mass deviation within 5 ppm of exact mass) and authentic standard. The boiling points and octanol-water partition coefficients (log*P*) values of aldehydes are enlisted for the purpose of comparing their distribution in OSOs

^2^Predicted boiling points are from Chemspider [34]. Reported boiling points are from a previous study [35]

^3^Log*P* are from Chemspider

^4^NR: Not reported

### Correlations of aldehydes and fatty acids with growth performance

**Fig. 4:**
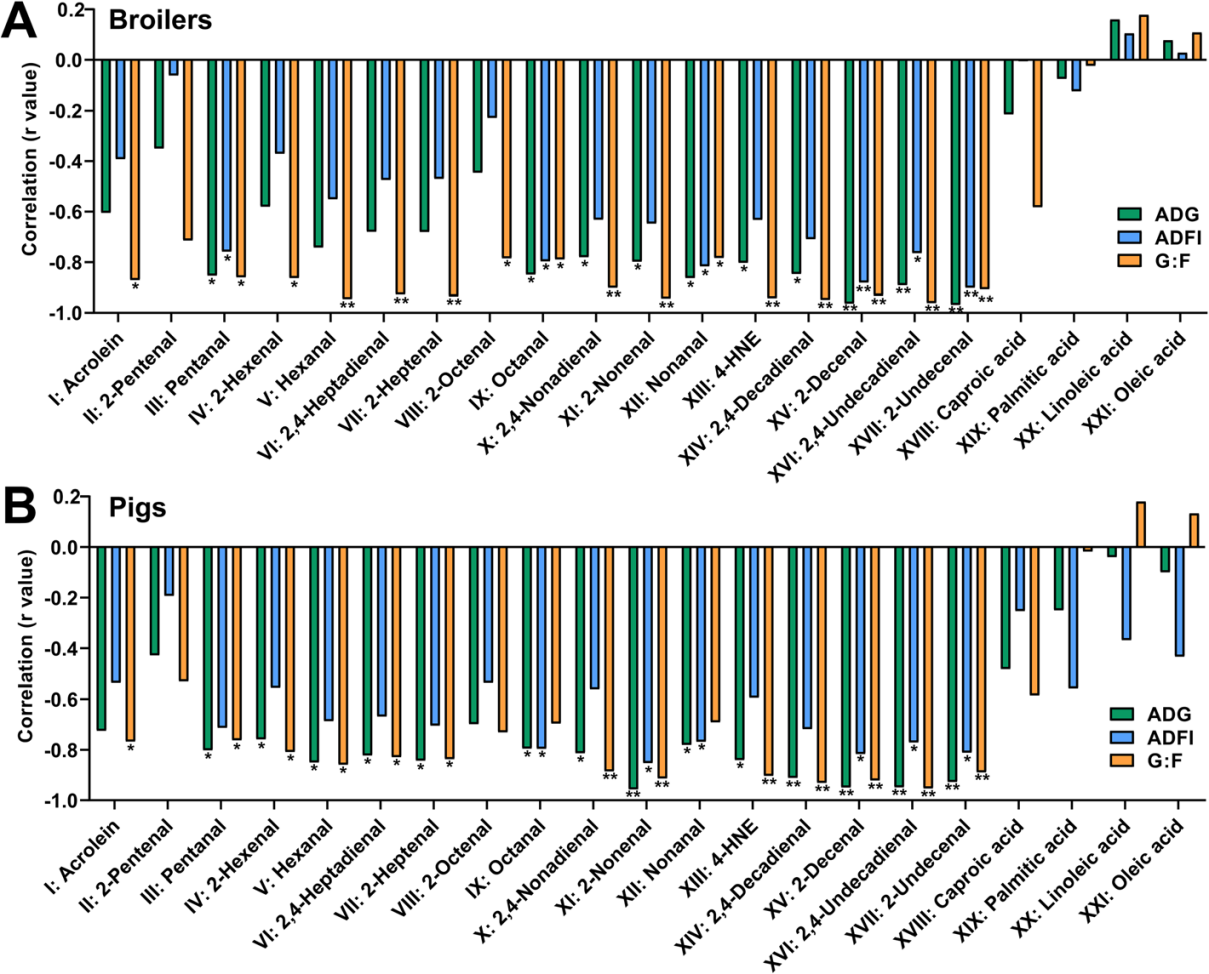
The Pearson correlations between aldehydes, fatty acids and growth performance of (**a**) broilers and (**b**) pigs. (*: *P* < 0.05; **: *P* < 0.01)

The values of individual aldehydes and fatty acids as potential prediction markers of growth performance were evaluated by their correlations with ADG, ADFI, and G:F. Interestingly, no significant correlations between individual fatty acids and animal growth performance in both broilers and pigs were observed (Fig. 4a and b). Majority of the 17 identified aldehydes, except for 2-pentenal, 2-octenal, octanal, and nonanal, had significantly inverse correlations with G:F; more than half had significant inverse correlations with ADG; and approximately one third had significantly inverse correlations with ADFI for both broilers and pigs (Fig. 4a and b). The C9-C11 unsaturated aldehydes, especially 2-decenal (XV) and 2-undecenal (XVII), had the highest negative correlations with ADG, ADFI, and G:F in both broilers and nursery pigs.

## Discussion

Diverse chemical and physical parameters have often been used as quality markers of edible oils. The compositional, sensory, and oxidative properties reflected by these quality markers are closely associated with nutritional value and toxicological profile of edible oils. Thermal treatments, such as chronic heat exposure and deep-frying, can dramatically alter the values of these quality markers, mainly through lipid oxidation and the formation of numerous degradation products. The current study examined the correlations of these quality markers, especially diverse aldehydes, with animal performance after feeding OSO to both broilers and pigs. The potential causes and significances of observed correlations are discussed based on the sources and functions of these markers.

### Correlations of common quality markers with growth performance

Within eight common indicators tested in this study, FFA, OFA, insoluble impurities, and AV had better correlations with growth performance than moisture, unsaponifiable matter, TBARS, and PV (Fig. 1a and b).

#### Moisture and unsaponifiable matter

The low correlations between moisture and unsaponifiable matter with animal performance in this study were expected, since these indicators mainly define the non-fatty acid impurities in oils and fats [36], and have poor relevance with the oxidative status of oils.

#### PV and TBARS

Though being widely used to define the extent of lipid oxidation in animal feed [27, 37, 38], PV and TBARS were poorly correlated with animal performance in the current study. This discrepancy could be attributed to the nature of PV and TBARS analysis. Hydroperoxides, the intermediates in lipid oxidation and the target molecules of PV analysis, are highly susceptible to further degradation to secondary LOP under thermal treatment [39]. Therefore, PV is not suitable for evaluating the quality of heavily-oxidized lipids, including several OSOs in the current study. Like hydroperoxides, MDA, a dialdehyde and the target of TBARS assay, is also highly reactive and unstable. Prolonged heating of vegetable oils and fatty acids, including oleic acid, linoleic acid, and linolenic acid, has been shown to decrease MDA concentration and TBARS value after peaking [31, 40, 41]. In addition, TBARS assay can be confounded by the color reactions between thiobarbituric acid and non-MDA components in the samples [42, 43]. All these issues limit the ability of TBARS to reflect the real oxidative status of vegetable oils, especially for the ones undergone severe thermal stress.

#### FFA

Free fatty acids can be formed as a consequence of hydrolysis reactions triggered by elevated temperature [4, 44]. The energy value of free fatty acids in animal feed has been evaluated in many studies without an agreement on their impacts. Negative effects of free fatty acids on digestible energy have been observed when feeding the oils with a range of free fatty acids content to both broilers and pigs [45–47]. However, in other studies, feeding oxidized lipids either did not affect or have little effect on nutrient digestibility [22, 38]. In the current study, FFA value measured by the AOCS method had significant inverse correlations with ADF and G:F in both broilers and pigs (Fig. 1a and b). Surprisingly, individual free fatty acid in CSO and OSOs had rather poor correlations with animal performance (Fig. 4a and b), mainly due to the fact that the concentrations of three major fatty acids, i.e. palmitic acid, oleic acid, and linoleic acid, were not increased by thermal treatments, except for the 225 °C-10.5 h treatment (Fig. 3s through u). Interestingly, caproic acid, a fatty acid known to contribute to rancidity [48], had greater inverse correlations with performance than palmitic acid, oleic acid, and linoleic acid (Fig. 4a and b). Further investigations are needed to examine the inconsistency between the FFA value and individual free fatty acids concentrations. One plausible cause is the low specificity of alkalimetric titration in the AOCS Official Method Ca 5a-40 that uses phenolphthalein as the indicator to determine the acidity of examined oil samples. It has been reported that, besides free fatty acids, polar components in oils, including phospholipids and many lipid oxidation products, are also alkali-titratable, and can interfere the accuracy of the measurement [49].

#### OFA and insoluble impurities

In the current study, OFA and insoluble impurities were both inversely correlated with animal performance in both broilers and pigs (Fig. 1a and b). The insoluble impurities analysis measures the insoluble content undissolved in kerosene and petroleum ether. Because the soybean oil tested in this study was commercially refined, insoluble plant mass and foreign substances were not expected to count for the bulk of measured values of insolubles. Instead, the polar compounds generated by thermal oxidation could be the major OSO components insoluble in kerosene and petroleum ether. The OFA analysis determines the post-saponification components that are insoluble in petroleum ether, but soluble in diethyl ether. By definition, oxidized fatty acids are mainly comprised of epoxy, hydroxy and keto acids from the decomposition of hydroperoxides [50]. Because both insoluble impurities and OFA analyses measure the components insoluble in petroleum ether, the two analyses are expected to have overlapping coverage on their targets in the OSOs. This fact can partially explain their comparable inverse correlations with growth performance of pigs and broilers in the current study. Nevertheless, both methods are proximate analysis and their values as predicators of animal performance are likely limited by the lack of specificity in their evaluation on the nutritional and toxicological properties of oxidized lipids.

#### AnV

AnV is an index of secondary LOP, reflecting the level of aldehydes, principally 2-alkenals and 2,4-dienals, in oils. Except for CSO and the 45 °C-336 h OSO, other OSOs in the current study had their AnV greater than 10, which is the cutoff value used by the industry for good refined oils. In the current study, AnV had the strongest inverse correlations with ADG, ADFI, and G:F ratio in both broilers and pigs among 8 examined common oil indicators, which is in contrast to many feeding studies that showed poor correlations between AnV and ADG, ADFI, or G:F [5, 16, 23, 51]. It is notable that the current study only used soybean oil for multiple thermal treatments and feeding groups while previous studies had different oils with few thermal treatments and feeding groups [5, 16, 23, 52]. In addition, young animals used in this study are likely more sensitive to oxidative stress than older animals used in previous studies [5, 23]. These differences may facilitate the observation of more robust correlations between AnV and performance in the current study. Nevertheless, the AnV test has its limitation. A confounding feature of the AnV test is that the intensity of *p*-anisidine colorimetric response depends on the unsaturation of aldehydes. Typically, alkadienals are more colorigenic than alkenals, followed by alkanls at identical concentrations [39]. Therefore, individual aldehydes can have different correlations with animal performance from AnV.

### Correlations of aldehydes with growth performance

Aldehydes in food or feed can be absorbed and metabolized *in vivo*, leading to oxidative stress and adverse health problems [53–56]. In addition, many aldehydes generated from lipid oxidation have relatively low odor threshold on rancidity, which could affect dietary palatability and reduce feed intake [8]. Therefore, the observation of inverse correlations between aldehydes and animal performance in this study was expected, and is consistent with the inverse correlation between AnV and animal performance. The more meaningful observation from this study was the robust inverse correlations of C9-C11 unsaturated aldehydes with ADG, ADFI, and G:F ratio of both broilers and pigs. Interestingly, a previous study has showed that C9-C10 aldehydes, including 2,4-decadienal, 2-decenal, HNE, 2,4-nonadienal, and 2-nonenal, had much lower LD_50_ values than C4-C8 aldehydes when comparing their cytotoxicity to rat hepatocytes [57]. All these observations imply that C9-C11 unsaturated aldehydes may contribute more to the toxicity and adverse effects of OSO than the aldehydes in shorter carbon chain. The factors that could contribute to this phenomenon, including concentration, bioavailability, and bioactivity of these C9-C11 aldehydes, are discussed as follows.

#### Concentration

Aldehyde toxicity is dose-dependent. The concentrations and profiles of individual aldehydes in thermally-oxidized oils are expected to be affected by fatty acid composition, thermal treatment, and boiling points of individual aldehydes. Soybean oil used in the current study is abundant in linoleic acid. Some of the most abundant aldehydes in the OSOs, including 2,4-decadienal (XIV), 2-octenal (VIII), pentanal (III), hexanal (V), and 4-HNE (XIII), are mainly formed by the homolytic β-scission of linoleic acid hydroperoxides, i.e. 9-hydroperoxy and 13-hydoperoxy linoleic acid [7, 12, 31]. Besides being affected by fatty acid composition, heating-elicited aldehyde formation is also time- and temperature-dependent. In general, higher heating temperature and longer heating time increase aldehyde formation in oils. However, excessive heating in temperature and time can decrease the levels of selective aldehydes in oils by degradation and evaporation [58]. The boiling points of various aldehydes in three edible oils have been evaluated previously [35]. In the current study, the boiling points of individual aldehydes largely match their distribution profile in five OSOs (Table 3). All C9-C11 aldehydes (X-XVII) peaked in the 135 °C-42 h and the 180 °C-21 h OSO treatments, while majority of C3-C8 aldehydes (I-IX) peaked in the 90 °C-84 h treatment. Moreover, the 225 °C-10.5 h treatment led to lower concentrations of aldehydes compared to the 135 °C-42 h and the 180 °C-21 h treatments.

#### Bioavailability

Systematic evaluation of the bioavailability of major aldehydes in edible oils has not been reported. However, aldehydes and other secondary LOP might have greater bioavailability than their hydroperoxide precursors based on: 1) linoleate hydroperoxide has low bioavailability because a substantial amount of radioactivity was retained in the gastrointestinal tract of rats after oral dosing of ^14^C-labeled methyl linoleate hydroperoxide [59]; 2) secondary LOP of linoleic acid is bioavailable because more than 50% radioactivity was recovered in urine after the oral administration of ^14^C-labeled secondary LOP in rats [53]; and 3) the absorption of specific aldehydes, including 2-nonenal and 2-pentenal, has been observed [60]. As for the OSO aldehydes in the current study, the absorption and metabolism events in the gastrointestinal tract and the liver determine their bioavailability in the two non-ruminant species used herein. The absorption of aldehydes is directly related to their solubility and membrane permeability, which are determined by carbon chain length and functional groups in the structures. Based on their log*P* values (Table 1), the membrane permeability of detected aldehydes should be sufficient for effective absorption, especially C9-C11 aldehydes (X-XVII) with the log*P* ranging from 2.65 to 4.23. In addition, extensive metabolism is expected to occur to OSO aldehydes because aldehyde dehydrogenases, alcohol dehydrogenases, and glutathione s-transferases are widely distributed in the stomach, small intestine, and liver [61]. Therefore, absorbed aldehydes are likely present in systemic circulation and post-hepatic tissues in the forms of their metabolites [8]. For example, after the oral administration of 2-nonenal to rats, stomach digesta had significant amount of its corresponding carboxylic acid, 2-nonenoic acid, while urine was enriched with its mercapturic acid metabolites [60].

#### Bioactivity

The bioactivity of aldehydes is greatly affected by their reactivity. A general reactivity of all aldehydes is from the electron-deficient carbon atom in the carbonyl group because it can readily react with primary amines, such as amino acids, to forming Schiff bases [12]. In *α*, *β*-unsaturated aldehydes, the presence of a carbon-carbon double bond that conjugates with the carbonyl group further increases the reactivity because the additional electrophilic *β* carbon in their structures can form covalent adducts through Michael addition [62]. As for 4-HNE, an oxygenated *α*, *β*-unsaturated aldehyde, the additional hydroxy group at *γ* carbon further increases the electrophilicity and reactivity of its *β* carbon. Hence, saturated, *α*, *β*-unsaturated, and oxygenated *α*, *β*-unsaturated aldehydes can react with diverse electron-rich biomolecules, including glutathione, proteins, and nucleic acids, to form covalent adducts [9, 63–65]. The direct and indirect consequences of these reactions, including oxidative stress, posttranslational modification of enzymes and proteins, and transcriptional regulation of gene expression, are expected to affect many metabolic and physiological processes that control growth and feed intake. Despite its importance, the reactivity is not the sole determining factor of the bioactivity of aldehydes, especially for their *in vivo* toxicity, because the reported no-observed-adverse-effect level (NOAEL) values of *α*, *β*-unsaturated aldehydes from the short-term toxicity tests did not show clear correlations between electrophilicty and toxicity [8]. In addition, among detected OSO aldehydes, acrolein and 4-HNE had the highest electrophilicity and reactivity [66], but no correlation was found between acrolein and the performance of growing and finishing pigs [23, 52], while those correlation was found in broilers from 4 to 25 d of age [51].

### Evaluation of aldehydes as prediction markers of animal performance

Among detected aldehydes in OSOs, hexanal, 2,4-decadienal, and 4-HNE, three aldehydes that are mainly derived from the peroxidation of n-6 fatty acids have been used for monitoring oil quality in animal nutrition studies [67]. In this study, the correlations of 2,4-decadienal and 4-HNE with animal performance were much greater than that of hexanal. Hexanal, as a saturated aldehyde, is both volatile and stable, and has been frequently served as an indicator of lipid oxidation, in headspace gas chromatography analysis [68]. However, hexanal has much lower cytotoxicity than 2,4-decadienal and 4-HNE [69, 70], and is not an ideal prediction marker of animal performance. As for 4-HNE and 2,4-decadienal, 4-HNE is one of the most cytotoxic secondary LOP [9, 12], while 2,4-decadienal was commonly detected as the most abundant aldehyde in the thermally-oxidized vegetable oils rich in linoleic acid [31, 35, 71]. Therefore, their robust inverse correlations with ADG were not surprising. Interestingly, other C9-C11 aldehydes, especially 2-decenal and 2-undecenal, had even better correlations with ADG, ADFI, and G:F ratio than 4-HNE and 2,4-decadienal in both broilers and pigs (Fig. 4a and b). Like other *α*, *β*-unsaturated aldehydes, 2-decenal and 2-undecenal are natural flavorants and food additives. Limited studies on their bioactivities have been conducted. Together with 2,4-decadienal, both 2-decenal and 2-undecenal are considered as safe food additives based on the results from rodent acute toxicity tests [72]. Nevertheless, 2,4-decadienal, 2-decenal, and 2-undecenal were also identified as the most active nematicidal compounds in *Ailanthus altissima* against the root knot nematode *Meloidogyne javanica* [73], suggesting their toxicity to eukaryotic species. More studies are needed to validate the observed correlations of 2-decenal and 2-undecenal with animal performance and to also examine the topics that could be associated with the correlations, such as whether their fatty and fruity type odor could negatively affect feed intake, and whether their relatively higher log*P* values could be translated to higher bioavailability to the active metabolic organs and tissues.

## Conclusions

In summary, AnV was ranked as the top indicator for predicting animal growth performance among eight common oil quality indicators. Subsequent profiling of aldehydes in OSOs through LC-MS-based chemometric analysis revealed robust correlations between C9-C11unsaturated aldehydes, especially 2-decenal and 2-undecenal, and animal performance. This finding provides a new opportunity to establish prediction markers in the feeding of oxidized lipids to non-ruminant animals.

## Supplementary information

**Additional file 1: Table S1.** Temperature variance and heating time of CSO to generate different OSOs. **Table S2.** Composition of experimental diets, as-is basis

## Data Availability

All data that support the findings of this study are included in this article.

## Abbreviations

ADFI: Average daily feed intake
ADG: Average daily gain
AnV: *p*-Anisidine value
AOCS: American Oil Chemists’ Society
BW: Body weight
CSO: Control soybean oil
DPDS: 2,2′-Dipyridyl disulfide
FFA: Free fatty acids
G:F: Gain to feed ratio
HCA: Hierarchical cluster analysis
4-HNE: 4-Hydroxynonenal
HQ: 2-Hydrazinoquinoline
I: Insoluble impurities
IACUC: Institutional Animal Care and Use Committee
LC-MS: Liquid chromatography-mass spectrometry
Log*P*: Octanol-water partition coefficients
LOP: Lipid oxidation products
M: Moisture
MDA: Malondialdehyde
MS/MS: Tandem MS
*m/z*: Mass-to-charge ratio
NOAEL: No-observed-adverse-effect level
NRC: National Research Council
OFA: Oxidized fatty acids
OSO: Oxidized soybean oil
PC: Principal component
PCA: Principal component analysis
PTAG: Polymerized tryacylglycerides
PV: Peroxide value
QTOFMS: Quadrupole time-of-flight mass spectrometer
SIC: Single-ion counts
TBARS: Thiobarbituric acid reactive substances
TIC: Total-ion counts
TPC: Total polar compounds
TPP: Triphenylphosphine
U: Unsaponifiable matter
UPLC: Ultra-performance liquid chromatography
