# Supplementary material for: Identification of C9-C11 unsaturated aldehydes as prediction markers of growth and feed intake for non-ruminant animals fed oxidized soybean oil
Source: J Anim Sci Biotechnol. 2020 May 8;11:49. doi: 10.1186/s40104-020-00451-4 (PMC7206673; doi:10.1186/s40104-020-00451-4)
Supplement: Supplementary file 1 — Additional file 1: Table S1. Temperature variance and heating time of CSO to generate different OSOs. Table S2. Composition of experimental diets, as-is basis [file 40104_2020_451_MOESM1_ESM.docx]

**Supplemental Tables**

**Table S1. Temperature variance and heating time of CSO to generate different OSO**

| Heating temperature, °C | Heating time, h |
| --- | --- |
| 22.5 (room temperature) | 0 |
| 46.1 ± 0.30 | 336 |
| 66.4 ± 1.58 | 168 |
| 89.0 ± 1.63 | 84 |
| 131.5 ± 3.56 | 42 |
| 170.5 ± 9.03 | 21 |
| 218.1 ± 11.39 | 10.5 |
| The temperatures in the thermal processes of preparing 45 °C-336 h, 67.5 °C-168 h, 90 °C-84 h, 135 °C-42 h, 180 °C-21 h, and 225 °C-10.5 h OSO were measured 11, 8, 4, 6, 21, and 21 times, respectively. | |

| **Table S2. Composition of experimental diets, as-is basis** | | | | |  |
| --- | --- | --- | --- | --- | --- |
| Item | Broiler | |  | Pig | |
|  | Phase 1, d 0 to 14 | Phase-2, d 15 to 28 |  | Day 0 to 25 | |
| Corn | 48.92 | 50.82 |  | 38.70 | |
| Soybean meal | 36.83 | 35.00 |  | 32.80 | |
| Distillers dried grains with solubles | 3.00 | 3.00 |  | 0.00 | |
| Dried whey | 0.00 | 0.00 |  | 15.00 | |
| Calcium carbonate | 1.36 | 1.36 |  | 1.23 | |
| Monocalium phosphate | 0.00 | 0.00 |  | 0.75 | |
| Dicalcium phosphate | 0.82 | 0.76 |  | 0.00 | |
| Vitamin-mineral premix^1,2^ | 0.75 | 0.75 |  | 0.16 | |
| Sodium chloride | 0.34 | 0.35 |  | 0.40 | |
| *L*-Lysine·HCl | 0.13 | 0.13 |  | 0.48 | |
| *DL*-Methionine | 0.28 | 0.26 |  | 0.24 | |
| *L*-Threonine | 0.03 | 0.03 |  | 0.16 | |
| *L*-Valine | 0.00 | 0.00 |  | 0.08 | |
| Phytase^3^ | 0.04 | 0.04 |  | 0.00 | |
| Soybean oil^4^ | 7.50 | 7.50 |  | 10.00 | |
| Total | 100.00 | 100.00 |  | 100.00 | |
| Calculated composition |  |  |  |  | |
| ME, kcal/kg | 3,250 | 3,270 |  | 3,760 | |
| CP, % | 22.8 | 22.0 |  | 21.4 | |
| Lysine, % total | 1.34 | 1.29 |  | 1.58 | |
| Lysine, % digestible | 1.20 | 1.16 |  | 1.43 | |
| Methionine + Cysteine, % total | 0.99 | 0.95 |  | 0.89 | |
| Calcium, % total | 0.86 | 0.84 |  | 0.83 | |
| P, % digestible | 0.21 | 0.20 |  | 0.40 | |
| ^1^ For broilers, the vitamin premix provided the following per kilogram of diet: vitamin A, 7,275 IU; vitamin D_3_, 2,425 IU; vitamin E, 15.8 IU; menadione, 1.0 mg; choline, 1,290 mg; vitamin B_12_, 10 μg; biotin, 40 μg; folic acid, 1.2 mg; niacin, 36 mg; pantothenic acid, 9.7 mg; pyridoxine, 1.0 mg; riboflavin, 4.9 mg; thiamine, 1.2 mg; iron, 124 mg; manganese, 111 mg; zinc, 111 mg; copper, 11 mg; iodine, 800 μg; selenium, 0.22 mg.  ^2^ For pigs, the vitamin premix provided the following per kilogram of diet: vitamin A, 2,140 IU; vitamin D_3_, 245 IU; vitamin E, 17.5 IU; menadione, 1.0 mg; vitamin B_12_, 18 μg; niacin, 19.6 mg; pantothenic acid, 9.4 mg; riboflavin, 3.8 mg; iron, 99 mg; manganese, 23.4 mg; zinc, 99 mg; copper, 9.9 mg; iodine, 180 μg; selenium, 0.18 mg.  ^3^ Provided 1,000 phytase units per kg of complete feed (Ronozyme HiPhos GT 2700, 2,703,000 phytase units/kg, DSM Nutritional Products NA, Parsippany, NJ).  ^4^ Soybean oil included CSO or OSO. | | | | |  |
